# Editorial: Overcoming obstacles of cancer immunotherapy: the important role of emerging nanomedicine

**DOI:** 10.3389/fonc.2024.1406244

**Published:** 2024-04-05

**Authors:** Aimin Jiang, Wangxiao He, Yu Yao

**Affiliations:** ^1^ Department of Medical Oncology, The First Affiliated Hospital of Xi’an Jiaotong University, Xi’an, China; ^2^ Department of Talent Highland, The First Affiliated Hospital of Xi’an Jiao Tong University, Xi’an, China

**Keywords:** cancer, immunotherapy, immune checkpoint inhibitors, tumor immune microenvironment, nanomedicine

Targeting the immune system using checkpoint blockade antibodies and chimeric antigen receptor T-cell (CAR-T) therapy has proven to be a successful clinical approach for cancer immunotherapy (1). Despite significant advancements in this field, several persistent challenges remain that demand attention. In 2020, Hegde et al. delineated ten pivotal obstacles to cancer immunotherapy, spanning from uncertainties in translating pre-clinical findings to identifying the optimal combination therapies for specific patients (2). These challenges encompass enhancing treatment response rates, elucidating immune resistance mechanisms, conquering immunotherapy resistance, identifying and managing immune-related adverse events (irAEs), identifying reliable predictive biomarkers, determining optimal combination strategies, and crafting personalized treatment regimens based on the unique characteristics of each patient (**Figure 1**). Nanomedicine has recently emerged as a potentially revolutionary domain for innovating therapeutic approaches, particularly in the realm of cancer detection and treatment (3). In recent years, a growing body of nanoparticles with distinct properties has been developed to overcome the hurdles of cancer immunotherapy (4–6). This Research Topic in *Frontiers in Oncology* provides insightful information about the critical role of nanomedicine in addressing the challenges of cancer immunotherapy.

**Figure 1:**
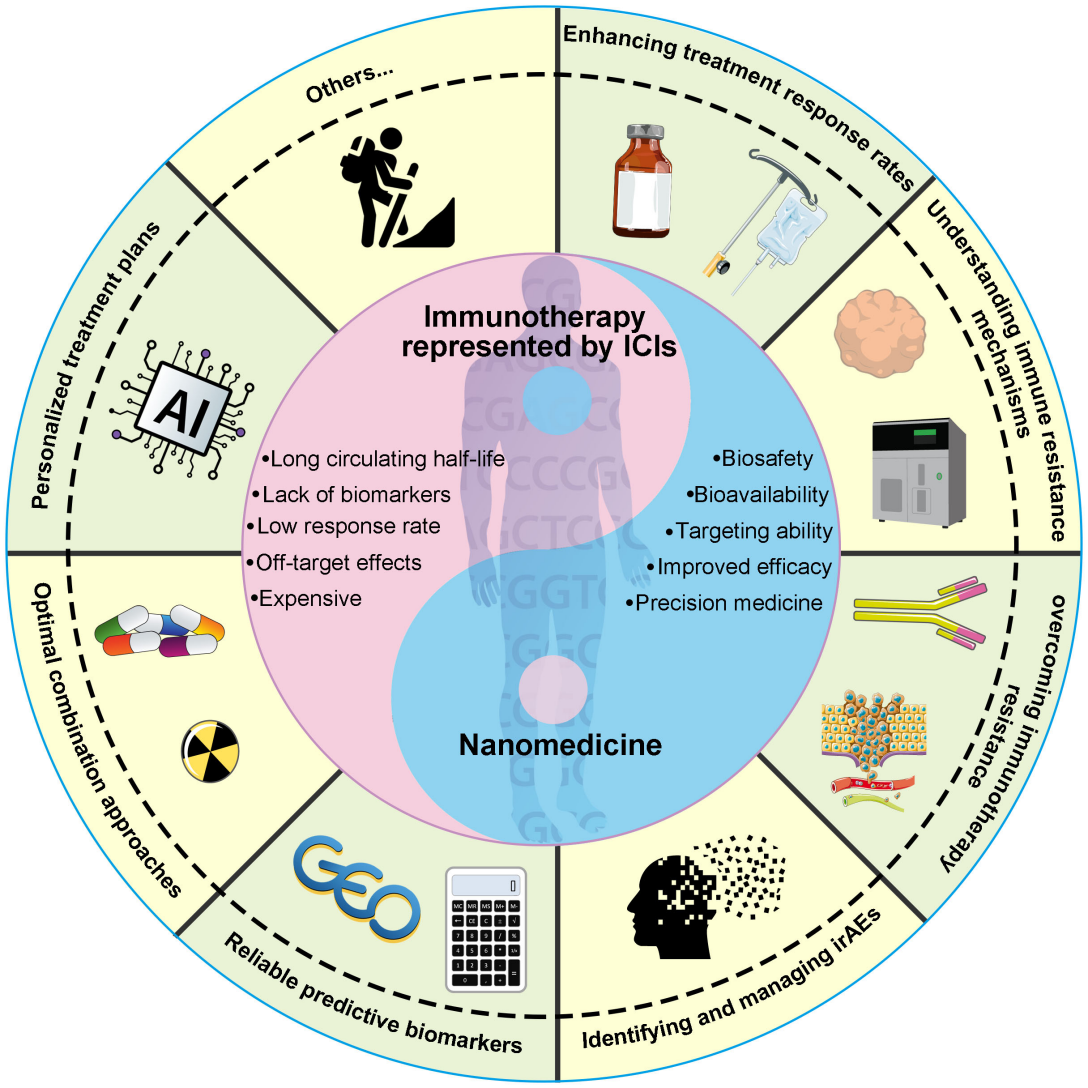
The challenges of cancer immunotherapy and the important role of emerging nanomedicine.

Cancer immunotherapy faces a significant challenge in the form of treatment resistance, posing a major hurdle to its effectiveness. Studies have shown that approximately 30% of non-small cell lung cancer (NSCLC) patients exhibit resistance to initial immunotherapy, whether utilizing immune checkpoint inhibitors (ICI) alone or in combination with another ICI (7–10). Shao et al. conducted a review to explore the mechanisms underlying immunotherapy resistance and outlined recent breakthroughs in nanomedicine designed to combat this resistance. Tumor heterogeneity and complexity, characterized by intricate metabolic, inflammatory, and neovascular pathways, remain poorly understood yet prevalent contributors to immunotherapy resistance (11). Currently, immunotherapy resistance is classified into intrinsic and extrinsic mechanisms (11). Intrinsic mechanisms entail alterations in anti-tumor immune response pathways and tumor cell signaling, resulting in an immunosuppressive tumor immune microenvironment (TIME) (11). Extrinsic factors primarily encompass components of the TIME, including immune checkpoint molecules, diverse immune cells, and cytokines (11). Systemic anti-tumor immune responses are hindered by this intricate network, thus leading to resistance to immunotherapy. Consequently, various nanomedicine strategies have emerged to target tumor cell-associated signaling pathways and the suppressive TIME, with the aim of bolstering the anti-tumor immune response and enhancing the efficacy of immunotherapy (12–15).

TIME plays pivotal roles in the development, metastasis, and treatment response of malignancies (16). Altering the inhibitory components of TIME is essential for improving the response to cancer immunotherapy and devising novel anti-tumor immunotherapeutic strategies (17). Gao et al. conducted a retrospective cohort study consisting of 68 NSCLC patients to investigate the effects of platinum-based neoadjuvant chemotherapy (NACT) on the components and status of TIME. The authors collected the clinicopathological characteristics and paired tissue samples before and after NACT. Using histopathological techniques, they detected STING, PD-L1, and IFN-β expression levels, as well as the infiltration level of tumor-infiltrating lymphocytes (TILs). They revealed that NACT activated the STING/IFN-β pathway, increased the infiltration of CD3^+^ and CD8^+^ TILs, and upregulated PD-L1 expression. Furthermore, patients with ypTNM I, ypN0-1, and elevated CD3^+^ TILs after NACT experienced prolonged disease-free survival (DFS), while patients with ypN0 and elevated CD3^+^ TILs after NACT had better overall survival (OS) benefits. Nonetheless, given the limitations of the sample size and the methods employed for analyzing TILs infiltration, well-designed large-scale multicenter cohort studies are necessary in the future to validate these findings.

Zhang et al. conducted a review of recent advancements in lung cancer therapeutics from the lens of nanomedicine. They discussed strategies for overcoming hurdles in lung cancer immunotherapy, aiming to enhance overall treatment response and minimize treatment-related adverse events by improving the tumor-targeting properties of nanoparticles. Song et al. provided an overview of the latest research trends in nanotechnology for cervical cancer. Through a systematic review and bibliometric analysis of 997 relevant publications, they observed that the majority of research originated from China, with the Chinese Academy of Sciences emerging as the most prolific institution. Additionally, they noted that 60% of the top 10 institutions in terms of publication volume were from China. This indicates a significant interest in utilizing nanotechnology for cervical cancer research. The author also suggested that future research trend may involve integrating nanotechnology with ‘photothermal therapy’ and ‘indocyanine green’.

In the realm of biomimetic nanomedicine, surface modification of nanocarriers with biological membranes has attracted a lot of attention lately. This innovative approach preserves the intrinsic cellular characteristics, endowing nanoparticles with remarkable abilities such as immune evasion and targeted accumulation at tumor sites (18–20). Zhou et al. reviewed recent advances in the use of cancer cell membrane-coated nanoparticles (CM-NPs) for the diagnosis and treatment of breast cancer. CM-NPs are capable of evading immune surveillance, as well as possessing excellent penetration and targeting properties. They concluded that CM-NPs hold great potential as a pivotal drug delivery strategy in the diagnosis and treatment of various malignancies, including breast cancer. Recent studies have revealed that biomimetic nanoparticles derived from erythrocyte membranes exhibit excellent tumor-targeting capabilities by altering the endocytosis mechanism (6, 21). A recent study has introduced an innovative approach by developing an erythrocyte membrane biomimetic ICI proteinoid to selectively inhibit tumor cell PD-L1 via a distinct endocytosis pathway between cancerous and non-cancerous cells (6). *In vivo* experiments have shown that the biomimetic ICI proteinoid not only reactivates the anti-tumor immune response in a murine model of lung adenocarcinoma with coexisting idiopathic pulmonary fibrosis but also demonstrates a favorable safety profile by effectively eliminating any irAEs. This breakthrough broadens the boundaries of immunotherapy in lung cancer treatment (6).

In conclusion, this Research Topic provides an overview of the current progress and future challenges of nanotechnology in cancer immunotherapy. Nanomedicine, as a paradigm of precision medicine, holds great promise in addressing the hurdles of cancer immunotherapy from various perspectives.

## Author contributions

AJ: Conceptualization, Writing – original draft, Writing – review & editing. WH: Writing – review & editing. YY: Project administration, Supervision, Writing – review & editing.
